# Association Between Serum Vitamin D Levels and Frequency of Relapses in Patients With Multiple Sclerosis

**DOI:** 10.7759/cureus.14383

**Published:** 2021-04-09

**Authors:** Farah Mansoor, Vikash Kumar, Suneel Kumar, Navneet Kaur, Sidra Naz, Simra Shahid, Faryal Anees, Sidra Memon, Amber Rizwan

**Affiliations:** 1 Internal Medicine, Jinnah Sindh Medical University, Karachi, PAK; 2 Neurology, Ghulam Muhammad Mahar Medical College, Sukkur, PAK; 3 Internal Medicine, Adesh institute of Medical Sciences and Research, Bathinda, IND; 4 Internal Medicine, University of Health Sciences, Lahore, PAK; 5 Medicine, Jinnah Sindh Medical University, Karachi, PAK; 6 Obstetrics and Gynecology, Agha Khan University Hospital, Karachi, PAK; 7 Family Medicine, Jinnah Post Graduate Medical Center, Karachi, PAK

**Keywords:** multiple sclerosis, vitamin d, association, relapses

## Abstract

Introduction

Multiple sclerosis (MS) is an immune-mediated inflammatory disease of the central nervous system affecting the myelin sheath of neurons with a wide range of symptoms. Among various risk factors studied that can increase the relapse, vitamin D is also a potential risk factor. In this study, we will determine the association between vitamin D status and frequency of relapses in patients with MS.

Material and methods

Seventy-four (74) patients with a confirmed diagnosis of MS, with more than one (01) relapse per year, for a minimum of two years, were included in the case group. Seventy-four (74) participants with a confirmed diagnosis of MS with one (01) or no relapse per year, for a minimum of two years, were included in the control group. After informed consent, the patient blood was drawn via phlebotomy and was sent to the lab for vitamin D levels.

Results

The mean serum vitamin D level was significantly lower in case group compared to control group (18.21 ± 4.21 ng/mL vs. 29.21 ± 5.72 ng/mL; p-value: < 0.0001). The number of participants with vitamin D level less than 30 ng/mL were significantly higher in patients with case group compared to control group (78.37% vs. 50.0%; p-value: 0.0003)

Conclusion

In this study, patients with more relapses per year had low level of serum vitamin D. There is emerging strong evidence that vitamin D plays an important role in the pathogenesis, progression, and disease burden of autoimmune disease, including MS.

## Introduction

Multiple sclerosis (MS) is an immune-mediated inflammatory disease of the central nervous system affecting the myelin sheath of neurons with a wide range of symptoms. The prevalence of MS varies from high levels in North America and Europe (>100/100,000 inhabitants) to low levels in Eastern Asia and sub-Saharan Africa (2/100,000 population) [1]. The sensory symptoms and fatigue occur early in the disease course; while loss of mobility, fatigue, bowel/bladder dysfunction and spasticity are more commonly reported in later stage of disease [2]. The age of onset most commonly can range from 15 to 50 years and females are commonly affected [3]. 

Primarily, there are two forms of MS: relapsing and progressive [4]. Relapses pose numerous challenges to health care providers in the management of MS, and they can also significantly increase the cost of management of MS and decrease the quality of life of patients [5,6]. It is important to identify various risks associated with increased risk of relapse and mitigate them to reduce the frequency of relapses [7]. Various risk factors such as age, bacterial infection, vitamin D levels, sex, and pregnancy have been discussed in various studies as potential risk factors for relapses [8]. In this study, we will determine the association between Vitamin D status and frequency of relapses in patients with MS in local setting.

## Materials and methods

This case-control was conducted in the neurology department of various tertiary care hospitals from November 2017 to May 2020. Seventy-four (74) patients with a confirmed diagnosis of MS, with more than one (01) relapse per year, for a minimum of two years, were included in the case group. As a reference, seventy-four (74) participants with a confirmed diagnosis of MS with one (01) or no relapse per year, for a minimum of two years, were included in the control group. Participants taking vitamin D supplements were excluded from the study. MS was confirmed with magnetic resonance imaging (MRI), clinical symptoms, and cerebrospinal fluid analysis. Relapse was defined as a neurologic deficit associated with an acute inflammatory demyelinating event that lasts at least 24 hours in the absence of fever and infection [9]. Patient extended disability status scale (EDSS) was noted. After informed consent, the patient blood was drawn via phlebotomy and was sent to the lab for Vitamin D levels. Patients with vitamin D levels of less than 30 ng/ml were classified as Hypovitaminosis [10].

Statistical analysis was done using SPSS v. 22.0 (IBM Corporation, Armonk, NY, United States). Continuous variables, including age, duration of disease, number of relapses and mean vitamin D levels, were presented as mean and standard deviation. Categorical variables, including gender, treatment, and hypovitaminosis, were presented as percentages and frequencies. An Independent T-test was applied to compare the numerical values. Chi-square was applied to compare categorical data between the two groups. A p-value of less than 0.05 represented a difference between the case and control group and the null hypothesis was void.

## Results

The mean age of participants in the case group was 38 ± 6 years and for the control group, it was 37 ± 5 years. The EDSS score was 3.21 ± 1.02 for the case group and 3.11 ± 1.11 for the control group. However, differences between the two groups for age, time since diagnosis, EDSS score, were non-significant. The number of relapses in the case group was significantly higher compared to the control group (Table 1).

**Table 1:** Characteristics of case and control group. CD-20; B-lymphocyte antigen CD20.

Characteristics	Case group (n = 74)	Control group (n = 74)	p-value
Age (in years)	38 ± 6	37 ± 5	0.27
Time since diagnosis (years)	4.4 ± 2.1	4.2 ± 2.4	0.59
Female (%)	49 (66.2)	50 (67.5)	0.86
Extended disability status scale score (mean ± SD)	3.21 ± 1.02	3.11 ± 1.11	0.56
No of relapse per year (mean ± SD)	1.31 ± 0.58	0.52 ± 0.21	<0.0001
Current treatment			
Steroids	31 (41.9)	28 (37.8)	0.56
Interferon	23 (31.1)	20 (27.0)	
Anti-CD-20 therapy	20 (27.0)	26 (35.1)	

The mean vitamin D level was significantly lower in case group compared to control group (18.21 ± 4.21 ng/mL vs. 29.21 ± 5.72 ng/mL; p-value: < 0.0001). The number of participants with vitamin D level less than 30 ng/mL were significantly higher in patients with case group compared to control group (78.37% vs. 50.0%; p-value: 0.0003) (Table 2).

**Table 2:** Vitamin D status in case and control group.

Vitamin D level	Case group (n = 74)	Control group (n = 74)	p-value
Mean value (ng/mL)	18.21 ± 4.21	29.21 ± 5.72	< 0.0001
Hypovitaminosis (%)	58 (78.37)	37 (50.0)	0.0003

## Discussion

In our study, patients of MS with more than one relapse per year had lower level of serum vitamin D level compared to patients of MS with less than one relapse per year. The case group also reported a higher prevalence of hypovitaminosis compared to control group. 

Prior studies have shown that vitamin D levels are known to play a major role in MS. Munger et al. conducted a study in which the white participants proved the correlation between increased levels of 25-hydroxyvitamin D and decreased risk of MS; however, the blacks and Hispanics in the same study showed no significant relation between MS and vitamin D [11].

Various studies point out that vitamin D may not only be an environmental risk factor for the development of MS but may also play an important role in modulating disease activity and progression. Various studies point out that low vitamin D levels have been associated with increase relapse rate, like our study [12]. Low vitamin D level may also be responsible for increase disability and increase lesion load on magnetic resonance imaging (MRI) [13,14]. In recent years, numerous studies have been done to investigate if there is any role of vitamin D supplements in management of MS; however, based on systemic review done in 2020, the results are contradictory. Few studies show the benefit of Vitamin D supplements in reducing disease burden in MS patients and while few studies suggest otherwise [15].

However not every study has reported association between vitamin D levels and MS. Fragoso et al. in his study demonstrated that MS patients and control group had no significant differences between their vitamin D levels [16]. Some studies, focused on the disease activity, proved no interconnection between vitamin D and MS. Rito et al. in his study showed that there is no link between EDSS scores and vitamin D levels [17]. 

Vitamin D levels have been known to play particularly important role in other autoimmune diseases as well. Some studies have specified that the low vitamin D levels may be an important factor in the development of autoimmune diseases as several epidemiological studies have stated that there is a strong correlation between insufficient vitamin D and incidence of autoimmune diseases, like type 1 diabetes mellitus (T1D), systemic lupus erythematosus (SLE), rheumatoid arthritis (RA) and inflammatory bowel disease (IBD) [18,19]. In animal models for T1D, MS, SLE, IBD, and autoimmune uveitis, calcitriol proved to either avoid or enhance autoimmunity. Studies that dealt with vitamin D deficient animals proved increased inflammatory response and vulnerability to T1D and Crohn’s disease and were prone to be invaded by bacteria and infections [20].

To the best of our knowledge, this is the first study from Pakistan that studies the association of vitamin D level and frequency of relapses in MS patients. The study was conducted in various hospitals, allowing the sample to be diverse. However, further large-scale studies are needed to study Vitamin D level influence on various aspects of MS disease, including relapses, disability, and MRI lesion loads. If proven effective in local setting, Vitamin D may provide a cheap option in addition to existing therapies, to reduce relapses, disability, and disease burden, which may reduce the overall cost of management of MS and improve quality of life in MS patients.

## Conclusions

In this study, patients with more relapses per year had low level of serum vitamin D. There is emerging strong evidence that vitamin D plays an important role in the pathogenesis, progression, and disease burden of autoimmune disease, including MS. Therefore, it is important that various investigatory trials should be done to understand the role of vitamin D in MS pathogenesis and to determine its role in the management of MS.
